# PD-L1 in pancreatic ductal adenocarcinoma: a retrospective analysis of 373 Chinese patients using an in vitro diagnostic assay

**DOI:** 10.1186/s13000-017-0678-4

**Published:** 2018-01-17

**Authors:** Xiaolong Liang, Jian Sun, Huanwen Wu, Yufeng Luo, Lili Wang, Junliang Lu, Zhiwen Zhang, Junchao Guo, Zhiyong Liang, Tonghua Liu

**Affiliations:** 1 Department of Pathology, Peking Union Medical College Hospital, Peking Union Medical College and Chinese Academy of Medical Sciences, No. 1 Shuai Fu Yuan, Wangfujing, Beijing, 100730 People’s Republic of China; 2 Department of General Surgery, Peking Union Medical College Hospital, Peking Union Medical College and Chinese Academy of Medical Sciences, No. 1 Shuai Fu Yuan, Wangfujing, Beijing, 100730 People’s Republic of China

**Keywords:** Pancreatic, PD-L1, Immunohistochemistry, Prognosis

## Abstract

**Background:**

Programmed death ligand 1 (PD-L1) has shown potential as a therapeutic target in numerous solid tumors. Its prognostic significance has also been established in pancreatic ductal adenocarcinoma (PDAC). The present study aimed to explore PD-L1 expression in PDAC cases in a large Chinese cohort using an in vitro diagnostic (IVD) assay to provide further insight into the potential value of programmed cell death protein 1 (PD-1) as a therapeutic target.

**Methods:**

Three hundred seventy-three PDAC patients were retrospectively recruited in this study. Tissue microarray (TMA) blocks were made from available formalin-fixed and paraffin-embedded (FFPE) tumor and matched adjacent tissue specimens. We evaluated PD-L1 protein expression via immunohistochemistry (IHC) using a U.S. Food and Drug Administration (FDA)-approved IVD assay. The relationships between PD-L1 positivity and both clinicopathological characteristics and patient prognosis were analyzed. PD-1 expression and clinicopathological significance were also evaluated.

**Results:**

PD-L1 and PD-1 positivity were observed in 3.2% and 7.5% of cases, respectively. PD-L1 showed a predominantly membranous pattern in tumor cells, while no positive PD-L1 staining was observed in normal regions. Statistical analyses revealed that PD-L1 expression was associated with lymph node metastasis. PD-L1 positivity was a prognostic indicator of progression-free survival (PFS) and overall survival (OS) in univariate analyses, but only PFS remained statistically significant in multivariate analysis. PD-1 expression was detected in lymphocytes and was not associated with any clinicopathological feature except a history of pancreatitis.

**Conclusions:**

The PD-L1 positivity rate is low in PDAC when evaluated using a companion diagnostic assay. It remains an independent prognostic factor for poor PFS.

**Electronic supplementary material:**

The online version of this article (10.1186/s13000-017-0678-4) contains supplementary material, which is available to authorized users.

## Background

Pancreatic ductal adenocarcinoma (PDAC) is one of the deadliest malignancies of the digestive system. Presently, the median survival for PDAC patients is measured in months; and the five-year survival for the unstratified patient group is less than 5% [1] despite radical surgery and chemotherapeutic regimens. Therefore, novel therapeutic strategies to tackle this drastic situation are urgently needed.

Programmed cell death protein 1 (PD-1), also known as B7–1, and its ligand, programmed death ligand 1 (PD-L1), also known as B7-H1, constitute a pair of corresponding regulatory receptors on the membrane of T cells and tumor cells, respectively. The role of the PD-1/PD-L1 pathway in the immune system, where it suppresses T cell activation and results in the loss of inhibitory function against tumors has been well elucidated and was recently summarized in a review by Vassiliki [2]. Meanwhile, the PD1/PD-L1 pathway and its relationship with cancer development and clinical outcomes have also been studied extensively [3, 4]. Moreover, blocking PD1/PD-L1 signaling has shown promising results in a broad spectrum of solid tumors, including pancreatic cancer [5, 6].

Analyses of PD-L1 expression in pancreatic cancer have yielded drastically variable results [7–12]. The expression rates range from 12.5% using a 5% cut-off point to 100%, where 60% to 90% of the tumor cells in each specimen were positive [13]. While the existing data suggest that PD-L1 is an indicator of an unfavorable prognosis, the association between PD-L1 positivity and clinicopathological parameters is less consistent [7, 10–12]. Further, most previous studies have adopted various but low cut-off values (ranging from 5%–10%) for determination of PD-L1 positivity, which has raised concerns about the validity of PD-L1 as a potential therapeutic target in PDAC.

In the present study, we aimed to assess the expression of PD-L1 in PDAC and its relationship with patient outcomes in a large Chinese cohort. We adopted a U.S. Food and Drug Administration (FDA)-approved in vitro diagnostics (IVD) assay (Roche SP263) and a manufacturer-validated cut-off value (25%) to provide additional information for the potential value of PD-L1 as a therapeutic target in PDAC patients. Meanwhile, PD-1 expression levels in PDAC tissue were also investigated.

## Methods

### Patient enrollment

A consecutive series of patients who underwent surgical resection of a pancreatic mass at Peking Union Medical College Hospital (PUMCH) from September 2008 to July 2014 with a histologically established diagnosis of PDAC based on the WHO 2010 classification [14] were retrospectively recruited in our study. Patient information, including gender, age, smoking history, alcohol consumption, previous medical history (diabetes mellitus and chronic pancreatitis), family history of PDAC, clinical symptoms, clinical staging, treatment received, progression-free survival (PFS), and overall survival (OS), were obtained from medical records. Pathological diagnoses were acquired from the pathology database. Clinical staging was based on the tumor-node-metastasis staging system outlined in the seventh edition of the American Joint Commission on Cancer [15]. PFS was defined as the period from the time of surgery to the establishment of tumor recurrence by medical imaging. OS was defined as the period from the time of surgery to patient death.

All subjects gave consent at the time of clinical intervention for future use of data/material for research purposes. Obtaining additional informed consent was not required for this retrospective study. The present study was approved by the Institutional Review Board of Peking Union Medical College Hospital (No. S-K118).

### Tissue microarray (TMA)

Representative, formalin-fixed, paraffin-embedded (FFPE) tissue blocks from the enrolled cases were retrieved from the tissue sample library of the pathology department of PUMCH along with the corresponding archived H&E slides. Representative tumor and non-tumor regions were identified by an experienced pathologist, and the regions of interest were annotated in the tissue block as the donor sites. A one-millimeter core needle was used for puncture. The puncture, transfer, and planting of tissue cores to the receiver block were performed using a semi-automated TMA construction system (Quick-Ray UT-06, UNITMA, Korea) according to the manufacturer’s instructions.

### Immunohistochemistry (IHC)

Four-micrometer-thick TMA sections were made from the TMA blocks and mounted on microscope slides. For PD-L1, the IHC staining was carried out on a Ventana Benchmark XT autostainer (Ventana, Tucson, AZ, USA) using an FDA-approved assay (Roche, SP263) following the manufacturer’s manual, which was acquired from Roche Diagnostics Co. (Shanghai, China). For PD-1, the TMA slides were air dried, incubated at 60 degrees Celsius for 20 min, deparaffinized in xylene, and hydrolyzed in gradient alcohol. Antigen retrieval was accomplished through hyperbaric/microwave treatment in citric acid-EDTA at 95 degrees Celsius for 15 min. Non-specific epitopes were then blocked with 10% goat serum. PD-1 antibody (rabbit monoclonal) was purchased from Zhongshan Gold Bridge (Beijing, China).

### Interpretation of IHC results

For PD-L1, the percentage of tumor cells with membranous staining was documented; positivity was defined as ≥25% of tumor cells showing membranous staining [16], and the statistics at different cut-offs (5% and 10%) were also documented. PD-1 positivity was defined as membranous or cytoplasmic staining in ≥1% of the tumor-infiltrating lymphocytes (TILs) [17]. All slides were evaluated by two experienced pathologists who had no prior knowledge of the selected cases. When discordance in interpretation occurred, the examiners discussed the results until they reached an agreement.

### Validation of the TMA IHC assay for PD-L1 and PD-1 expression

Given the concerns about the representativeness of the TMA, we set up a validation assay for PD-L1 and PD-1. For PD-L1, all cases that were classified as PD-L1-positive and 10 PD-L1-negative cases were selected. For PD-1, donor blocks from seventeen PD-1-positive cases and twelve PD-1-negative cases were recruited, and whole sections were made. PD-L1 or PD-1 expression was probed under the conditions described above. For PD-L1, the percentage of tumor cells with membranous staining was documented for each whole section. Consistency between the TMA and whole section staining was measured using the kappa value at different cut-off points (5%, 10%, and 25%). For PD-1, the whole-section slides were interpreted using the 1% cut-off value; the kappa values for the TMA and the whole section are provided.

### Statistical analysis

All data were processed using SPSS software (version 20.0, IBM SPSS software). Numerical data were examined with a K-S test for Gaussian distribution. Student’s t-test was used to detect differences in normally distributed numeric parameters. Meanwhile, the means and standard deviations were calculated. For categorical data or numerical data that were not normally distributed, a chi-square test or Spearman rank correlation test was used to analyze the relationship. The prognostic data were analyzed using a Kaplan-Meier analysis and Cox Regression. Survival curves were plotted. Factors yielding a *p* < 0.1 in the univariate analysis or considered clinically important were included in the multivariate analysis for PFS and OS. Missing data were not included in the statistical calculation. A *p* < 0.05 was considered statistically significant.

## Results

### Demographic characteristics of the patients

Three hundred seventy-three patients were evaluated in our study. Of these patients, 215 males and 158 females were included. The median age was 61 years, ranging from 29 to 82 years. In total, 132 and 65 patients had histories of smoking and alcohol consumption, respectively. Only one patient had a history of pancreatitis, and five patients reported a family history of PDAC. White blood cell count, differentiation, and lymph node metastasis were also evaluated. The demographic characteristics of the enrolled patients are summarized in Table 1.

**Table 1:** Association between clinicopathological parameters and PD-L1 expression in 373 PDAC patients

Clinicopathological characteristics	Total	PD-1		*p* value	PD-L1		*p* value
		Positive	Negative		Positive	Negative	
total	373	27	346		12	361	
Age (years)	373			0.924			0.550
Mean ± SD		60.12 ± 9.96	59.93 ± 9.39		58.42 ± 8.38	60.16 ± 9.96	
Gender				0.561			0.067
male	215	17	198		10	205	
female	158	10	148		2	156	
Smoking				0.568			0.772
yes	132	11	121		4	128	
no	238	16	222		6	232	
Drinking				0.236			0.139
yes	65	7	58		0	65	
no	305	20	285		10	295	
History of pancreatitis				**0.001**			0.868
yes	1	1	0		0	1	
no	370	26	344		10	360	
Family history				0.528			0.371
yes	5	0	5		0	5	
no	366	27	339		10	356	
WBC (×10^9^)				0.891			0.750
>10	30	2	28		1	29	
4–10	245	21	224		6	239	
<4	19	2	17		1	18	
Tumor differentiation				0.150			0.820
moderate /poor	307	20	287		10	297	
well	59	7	52		1	58	
pT				0.494			0.586
1	2	0	2		0	2	
2	18	0	18		1	17	
3	340	26	314		10	330	
4	8	1	7		0	8	
Clinical staging				1.000			1.000
1	10	0	10		0	10	
2	329	25	304		11	318	
3	13	1	12		0	13	
4	15	1	14		0	15	
Lymph node metastasis				0.759			0.058
yes	192	13	179		8	184	
no	90	7	83		0	90	
Lymphovascular invasion				1.000			1.000
yes	28	2	26		0	28	
no	135	11	124		4	131	

### Expression of PD-L1/PD-1 in PDAC tissues

**Fig. 1:**
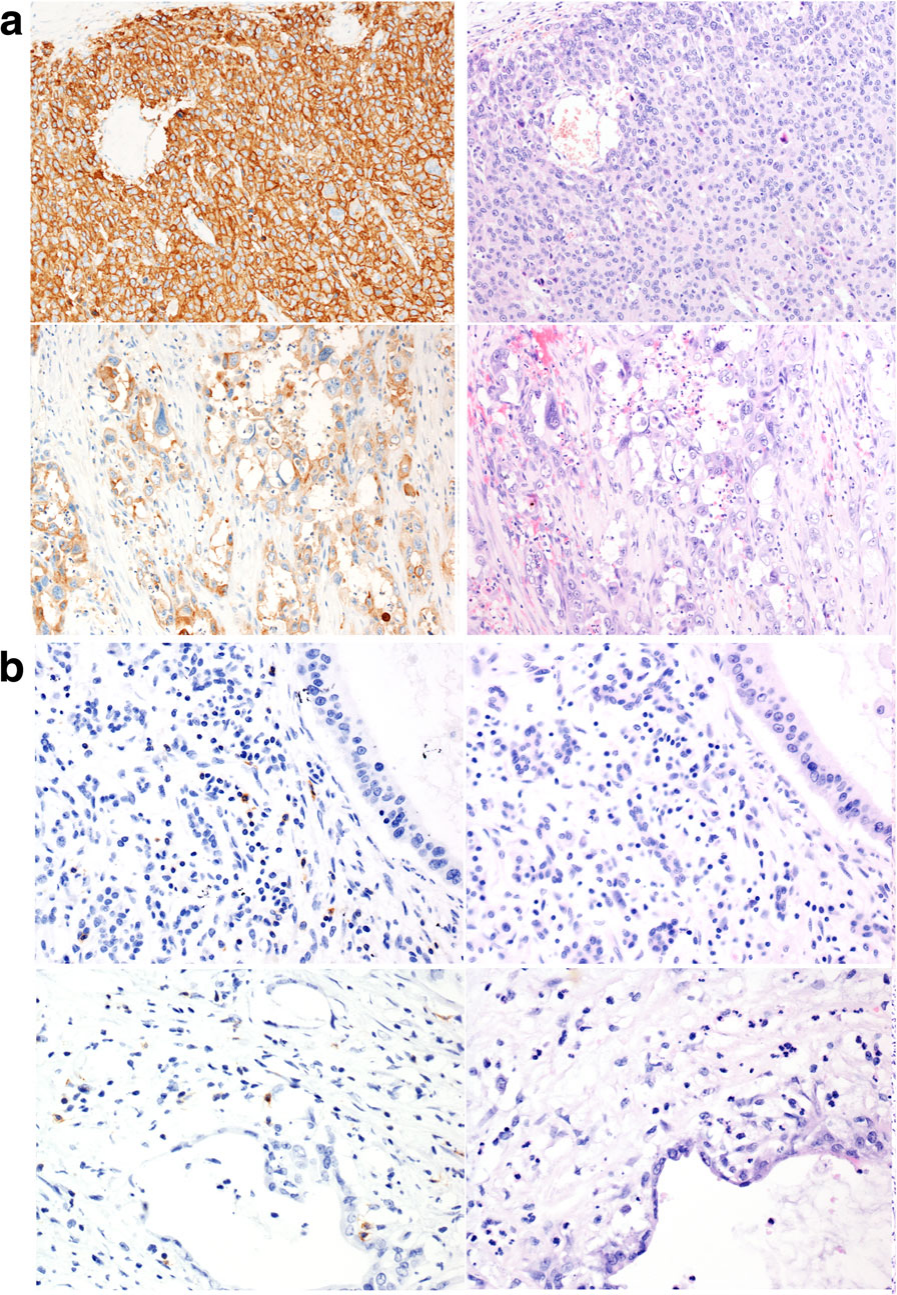
Exemplary figures for PD-L1 and PD-1 staining with corresponding H&E staining. **a** PD-L1 staining in PDAC tissue. PD-L1 staining was primarily observed on tumor cell membranes. Magnification, 10× objective. **b** PD-1 staining in PDAC tissue. PD-1 staining was primarily observed in tumor-infiltrating lymphocytes in the tissue. Magnification, 10× objective. Scale bars are equivalent to 100 μm

Of the 373 cases, 12 (3.2%) were positive for PD-L1 expression in cancer tissue using the 25% cut-off point. The number of PD-L1-positive cases increased to 22 (5.9%) and 33 (8.8%) when the cut-off points of 10% and 5%, respectively, were applied. However, no PD-L1 staining was observed in the normal tissue. Twenty-seven (7.5%) cases showed PD-1 expression in lymphocytes (Fig. 1). Moreover, no samples were positive for both PD-1 and PD-L1 at the same time.

### Association between PD-L1/PD-1 expression and clinicopathological parameters

No relationship was observed between the expression of PD-L1 and clinical features, such as gender, age, smoking, drinking, history of pancreatitis, or family history of PDAC. However, the association between PD-L1 expression and lymph node metastasis was marginally significant (*p* = 0.058). On the other hand, PD-1 expression was only related to a history of pancreatitis but not to any other parameters that we evaluated.

### PD-L1 expression was associated with PDAC patient prognosis

**Fig. 2:**
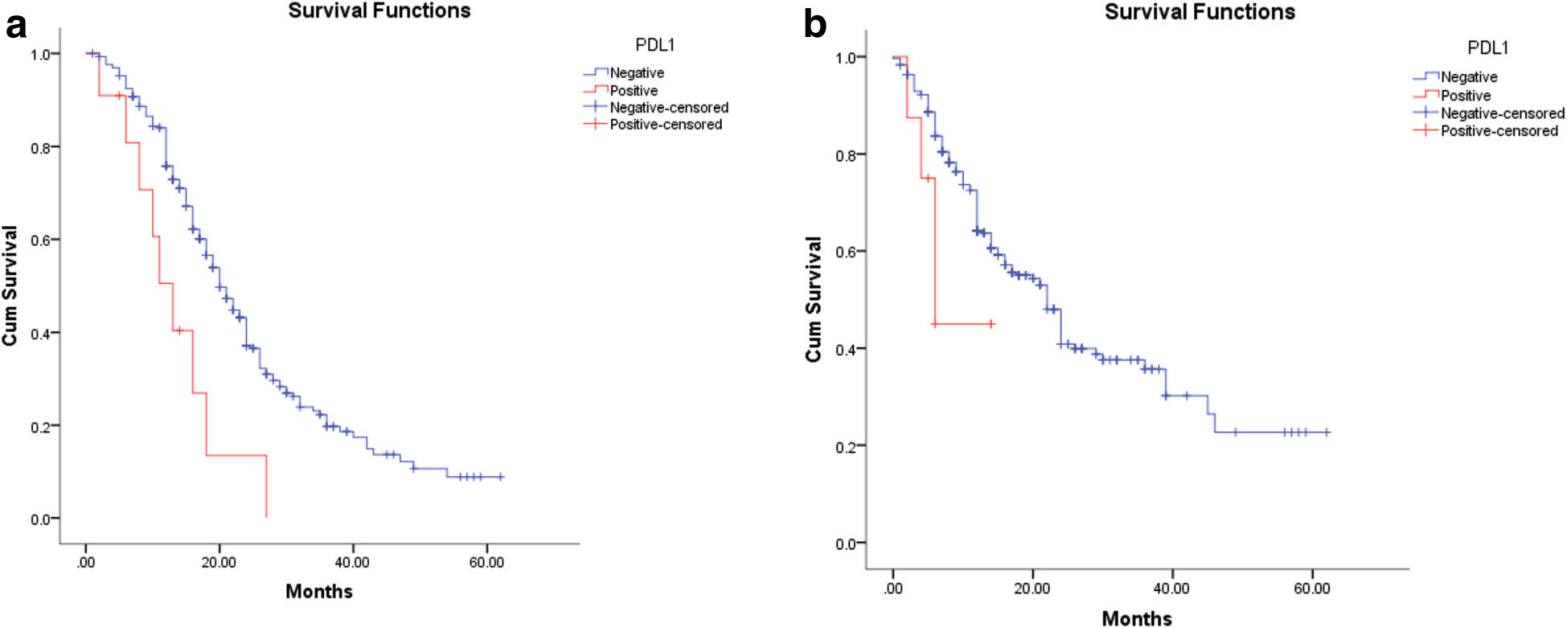
Kaplan-Meier plots of PD-L1 for OS and PFS. **a** Kaplan-Meier plot for OS showed that patients with PD-L1 positivity had significantly poorer OS than did PD-L1-negative individuals. **b** Kaplan-Meier plot for PFS showed that patients with PD-L1 positivity also had poorer PFS than did PD-L1-negative individuals. OS: overall survival; PFS: progression-free survival

Univariate analyses suggested that common bile duct invasion (*p* = 0.026), lymph node metastasis (*p* = 0.005), and PD-L1 expression (*p* = 0.003) were prognostic indicators of unfavorable PFS. The latter two factors remained statistically significant in multivariate analyses (lymph node metastasis, *p* = 0.012; PD-L1 positivity, *p* = 0.003). Meanwhile, lymph node metastasis (*p* = 0.001) and PD-L1 positivity (*p* = 0.002) were also prognostic indicators of poor OS in univariate analyses. However, only lymph node invasion retained statistical significance in multivariate analysis (*p* = 0.010) for OS, and PD-L1 positivity (*p* = 0.079) only showed a trend toward significance without reaching statistical significance (Table 2). In Kaplan-Meier tests, the PD-L1-positive arm showed significantly inferior OS and PFS than did the negative arm (Fig. 2). Using lower cut-off points narrowed the survival gap between the two arms, although the differences remained statistically significant (Additional file 1: Table S2).

**Table 2:** Univariate and multivariate analyses of prognostic factors for PFS and OS

Characteristics	p (Univariate analysis)		p (Multivariate analysis)	
	PFS	OS	PFS	OS
Gender	0.572	0.579		
Smoking	0.480	0.858		
Smoking index	0.347	0.183		
Drinking	0.780	0.208		
Diabetes mellitus	0.888	0.829		
Digestive presentation	0.895	0.060		0.125
WBC count	0.072	0.484	0.068	
Neoadjuvant chemotherapy	0.695	0.731		
Histological grading	0.074	0.082	0.125	0.089
Position	0.087	0.572	0.660	
Pancreatic capsule invasion	0.382	0.797		
Vascular invasion	0.350	0.270		
Common bile duct invasion	**0.026**	0.268	0.251	
Lymph node invasion	**0.005**	**0.001**	**0.012**	**0.010**
Neuroinvasion	0.809	0.218		
Post-operative chemotherapy	0.478	0.338		
PD-1	0.939	0.160		
PD-L1	**0.003**	**0.002**	**0.003**	0.079

PFS: progression free survival; OS: Overall survival; HR: Hazard ratio;

### Consistency of PD-L1/PD-1 staining between TMA and whole sections

Twenty-two and twenty-seven cases were included in the validation sets for PD-L1 and PD-1, respectively. The percentages of PD-L1-positive tumor cells in the TMA and in the corresponding whole sections are listed in Additional file 1: Table S3. At 25% and 10% cut-off points, one case (4.5%) that was negative in the TMA was found to be positive in the whole section. The number of inconsistent cases increased to 4 (18%) at the 5% cut-off point. The inconsistent cases were all positive in the whole sections but negative in the TMA. The kappa values of TMA and whole section samples at 5%, 10%, and 25% cut-off points were 0.621, 0.908, and 0.908, respectively. A related-samples Wilcoxon signed rank test yielded *p* = 0.281. PD-1 interpretations were 100% consistent in the TMA and whole sections (kappa = 1).

## Discussion

In the present study, we recruited 373 cases to evaluate the clinicopathological significance of PD-1/PD-L1 expression in PDAC. To the best of our knowledge, this is the largest PDAC cohort enrolled for evaluation of PD-1/PD-L1 expression.

Among the 373 patients recruited, only 12 (3.2%) were interpreted as PD-L1-positive according to the prespecified criteria. In the literature, the positive rates mainly ranged from 39.2% to 49.4% [8–10], although Lu et al. reported that 60% to 90% of tumor cells were positive in all 13 (100%) pancreatic cancer cases analyzed [13]. In contrast, in a study by Soares et al., the authors found that only 3 of 25 (12.5%) pancreatic cancer cases were positive for PD-L1 expression. In the present study, when the cut-off value was lowered to 5%, the corresponding positivity increased to 8.8%, which was quite comparable to that reported by Soares et al.

The difference in PD-L1 positivity rates in the literature and our study is only partly attributable to the different cut-off points used. Lu et al., who used an FDA-approved anti-PD-L1 monoclonal antibody (clone 28–8) in their study, suggested that the difference in positivity rates could be a result of variable antibody sensitivity and specificity [13]. In the present study, we adopted another FDA-approved in vitro diagnostic (IVD) assay for evaluation of PD-L1 expression in the PDAC cases. By using a standardized and fully automatic staining protocol following the pre-validated operating procedure provided by the manufacturer, our data tended to be quite reliable, and according to our validation set, replicable. Therefore, antibody quality could not seemingly be blamed for any differences. In fact, pancreatic cancer is considered rather nonimmunogenic [18], which was also supported by the sparse presentation of PD-1-positive (7.5%) TILs in our cases. The non-immunogenicity of pancreatic cancer explains the low positivity rate of T-cell regulators, such as PD-L1, in our study. In a recent study, exposure to an irradiated, granulocyte-macrophage-colony-stimulating factor (GM-CSF)–secreting, allogeneic PDAC vaccine significantly stimulated the expression of PD-L1 in tumor cells from PDAC patients [19]. Based on these findings, we postulate that immunogenic exposure in the individuals might have contributed to the differential expression of immunomodulators by tumor cells. Information about vaccination and patient allergy history could provide some insight into this issue, and further investigation is warranted.

On the other hand, applying a cut-off value of 5–10% means that even when more than 90% of the tumor cells do not express PD-L1, the case is still classified as PD-L1-positive. This raises concern about the efficacy of the marker to predict the PD-1/PD-L1 inhibitor response, although the lower cut-off points have well justified their usefulness in prognostic applications. As the assay adopted in the present study was developed in conjunction with therapeutic regimens for uroepithelial carcinomas only, our results might better reflect actual therapeutic indications for PD-L1 status than those of previous studies because a higher cut-off value was applied. In this regard, the most important value of the present study lies in the fact that we are alerting clinicians to the possible low percentage of PDAC patients who might be responsive to PD-1/PD-L1 inhibitor therapy. Regardless, such speculation requires further analyses in association with PDAC patient treatment schemes in future clinical trials.

PD-L1 has been found to be associated with poor prognosis in non-small cell lung cancer (NSCLC) [20], breast cancer [21], esophageal squamous cell carcinoma [22], and urothelial carcinoma [23]. PD-L1 positivity also predicted aggressiveness in melanoma [24] and recurrence in clear cell renal cell carcinoma [25].

In our cohort, survival analyses demonstrated that the prognosis of PD-L1-positive cases was significantly poorer than that of the PD-L1-negative cases regarding both PFS and OS (*p* < 0.05). Regretfully, PD-L1 was only an independent prognostic factor for PFS. When testing for the prognostic significance of PD-L1 for OS in multivariate analysis, the *p*-value did not reach statistical significance, likely due to the small number of PD-L1-positive cases.

The main limitation of the present study lies in the small area studied for each specimen, which was imposed by the TMA-based assay. Although PD-L1 heterogeneity has not been revealed in PDAC, it has been reported in several types of solid tumors [26, 27]. The representativeness of the TMA cores may thus raise certain concerns. To tackle this issue, we set up a validation set using representative whole sections. Our data suggested that when the 25% cut-off point was applied, only one of 22 (4.5%) cases had inconsistent results between the TMA and corresponding whole section. This finding suggests that the PD-L1 positivity rate could be underestimated in this study due to the small area that could be examined using TMA. However, such an underestimation may be limited to a relatively low extent. However, studies using more representative whole tissue blocks are desired.

## Conclusion

The PD-L1 positivity rate is low in PDAC when evaluated using a companion diagnostic assay. However, PD-L1 positivity remains an independent prognostic factor for poor PFS.

## Additional file

Additional file 1: **Table S1.** Association between clinicopathological parameters and PD-L1 expression using 10% or 5% cut-off points. **Table S2.** PFS and OS stratified by PD-L1 expression using different cut-off points. **Table S3.** Percentage of tumor cell with PD-L1 membranous staining in the validation set. (DOCX 21 kb)

## Abbreviations

FDA: Food and Drug Administration
FFPE: Formalin-fixed, paraffin-embedded
IHC: Immunohistochemistry
IVD: In vitro diagnostics
OS: Overall survival
PD-1: Programmed cell death protein 1
PDAC: Pancreatic ductal adenocarcinoma
PD-L1: Programmed death-ligand 1
PFS: Progression-free survival
PUMCH: Peking Union Medical College Hospital
TIL: Tumor-infiltrating lymphocyte
TMA: Tissue microarray
WHO: World Health Organization
