# Supplementary material for: PD-L1 in pancreatic ductal adenocarcinoma: a retrospective analysis of 373 Chinese patients using an in vitro diagnostic assay
Source: Diagn Pathol. 2018 Jan 17;13:5. doi: 10.1186/s13000-017-0678-4 (PMC6389094; doi:10.1186/s13000-017-0678-4)
Supplement: Supplementary file 1 — Table S1. Association between clinicopathological parameters and PD-L1 expression using 10% or 5% cut-off points. Table S2. PFS and OS stratified by PD-L1 expression using different cut-off points. Table S3. Percentage of tumor cell with PD-L1 membranous staining in the validation set. (DOCX 21 kb) [file 13000_2017_678_MOESM1_ESM.docx]

| Supplementary Table 1. Association between clinicopathological parameters and PD-L1 expression using 10% or 5% cut-off points | | | | | | | | | |
| --- | --- | --- | --- | --- | --- | --- | --- | --- | --- |
| Clinicopathological characteristics | Total | Cut-off=10% | | | P value | Cut-off=5% | | | P value |
|  |  | Positive | Negative | |  | Positive | | Negative |  |
| total | 373 | 22 | | 351 |  | | 33 | 340 |  |
| Age(years) | 373 |  | |  | 0.316 | |  |  | 0.481 |
| Mean±SD |  | 58.05±9.82 | | 60.23±9.91 |  | | 58.94±9.24 | 60.21±9.98 |  |
| Gender |  |  | |  | 0.140 | |  |  | 0.465 |
| male | 215 | 16 | | 199 |  | | 21 | 194 |  |
| Female | 158 | 6 | | 152 |  | | 12 | 146 |  |
| smoking |  |  | |  | 0.371 | |  |  | 0.713 |
| yes | 132 | 9 | | 123 |  | | 12 | 120 |  |
| no | 238 | 11 | | 227 |  | | 19 | 219 |  |
| Alcoholic consumption |  |  | |  | 0.224 | |  |  | 0.228 |
| yes | 65 | 1 | | 64 |  | | 3 | 62 |  |
| no | 305 | 19 | | 286 |  | | 28 | 277 |  |
| History of pancreatitis |  |  | |  | 1.000 | |  |  | 1.000 |
| yes | 1 | 0 | | 1 |  | | 0 | 1 |  |
| no | 370 | 20 | | 350 |  | | 31 | 339 |  |
| Family history |  |  | |  | 0.243 | |  |  | 0.058 |
| yes | 5 | 1 | | 4 |  | | 2 | 3 |  |
| no | 366 | 19 | | 347 |  | | 29 | 337 |  |
| WBC(×10^9^) |  |  | |  | 0.917 | |  |  | 0.172 |
| ＞10 | 30 | 1 | | 18 |  | | 2 | 17 |  |
| 4-10 | 245 | 12 | | 233 |  | | 17 | 228 |  |
| ＜4 | 19 | 2 | | 28 |  | | 5 | 25 |  |
| Tumor differentiation |  |  | |  | 0.651 | |  |  | 0.202 |
| moderate /poor | 307 | 18 | | 289 |  | | 29 | 278 |  |
| Well | 59 | 2 | | 57 |  | | 2 | 57 |  |
| Lymph node metastasis |  |  | |  | 0.759 | |  |  | 0.763 |
| yes | 192 | 13 | | 179 |  | | 17 | 175 |  |
| no | 90 | 4 | | 86 |  | | 7 | 83 |  |

| Supplementary Table 2. PFS and OS stratified by PD-L1 expression using different cut-off points | | | | | | |
| --- | --- | --- | --- | --- | --- | --- |
| Cut-off Point | PFS (months) | |  | OS(months) | |  |
|  | PD-L1(+) | PD-L1(-) | Log-rank p | PD-L1(+) | PD-L1(-) | Log-rank p |
| 25% | 11.17±1.90 | 24.10±1.15 | 0.003 | 13.15±2.45 | 24.905±1.17 | 0.002 |
| 10% | 13.83±1.89 | 24.22±1.16 | 0.016 | 15.03±2.03 | 25.14±1.20 | 0.003 |
| 5% | 15.85±1.85 | 24.42±1.20 | 0.015 | 16.92±1.66 | 25.20±1.22 | 0.018 |

Supplementary Table 3. Percentage of tumor cell with PD-L1 membranous staining in the validation set

| case | TMA | WHOLE SECTION |
| --- | --- | --- |
| **V001** | 0 | 0 |
| **V002** | 2 | 45 |
| **V003** | 50 | 45 |
| **V004** | 50 | 50 |
| **V005** | 30 | 35 |
| **V006** | 25 | 30 |
| **V007** | 60 | 55 |
| **V008** | 85 | 90 |
| **V009** | 60 | 50 |
| **V010** | 0 | 2 |
| **V011** | 1 | 5 |
| **V012** | 0 | 0 |
| **V013** | 60 | 55 |
| **V014** | 0 | 7 |
| **V015** | 0 | 5 |
| **V016** | 0 | 0 |
| **V017** | 0 | 0 |
| **V018** | 0 | 0 |
| **V019** | 50 | 60 |
| **V020** | 40 | 45 |
| **V021** | 90 | 90 |
| **V022** | 60 | 55 |
